# A Model of the Cardiorespiratory Response to Aerobic Exercise in Healthy and Heart Failure Conditions

**DOI:** 10.3389/fphys.2016.00189

**Published:** 2016-06-08

**Authors:** Libera Fresiello, Bart Meyns, Arianna Di Molfetta, Gianfranco Ferrari

**Affiliations:** ^1^Department of Clinical Cardiac Surgery, Katholieke Universiteit LeuvenLeuven, Belgium; ^2^Institute of Clinical Physiology, National Research CouncilRome, Italy; ^3^Medical and Surgical Department of Pediatric Cardiology, Bambino Gesù Children's HospitalRome, Italy

**Keywords:** modeling, cardiorespiratory, baroreflex, ventilation, gas exchanges, vasodilation, heart failure

## Abstract

The physiological response to physical exercise is now recognized as an important tool which can aid the diagnosis and treatment of cardiovascular diseases. This is due to the fact that several mechanisms are needed to accommodate a higher cardiac output and a higher oxygen delivery to tissues. The aim of the present work is to provide a fully closed loop cardiorespiratory simulator reproducing the main physiological mechanisms which arise during aerobic exercise. The simulator also provides a representation of the impairments of these mechanisms in heart failure condition and their effect on limiting exercise capacity. The simulator consists of a cardiovascular model including the left and right heart, pulmonary and systemic circulations. This latter is split into exercising and non-exercising regions and is controlled by the baroreflex and metabolic mechanisms. In addition, the simulator includes a respiratory model reproducing the gas exchange in lungs and tissues, the ventilation control and the effects of its mechanics on the cardiovascular system. The simulator was tested and compared to the data in the literature at three different workloads whilst cycling (25, 49 and 73 watts). The results show that the simulator is able to reproduce the response to exercise in terms of: heart rate (from 67 to 134 bpm), cardiac output (from 5.3 to 10.2 l/min), leg blood flow (from 0.7 to 3.0 l/min), peripheral resistance (from 0.9 to 0.5 mmHg/(cm^3^/s)), central arteriovenous oxygen difference (from 4.5 to 10.8 ml/dl) and ventilation (6.1–25.5 l/min). The simulator was further adapted to reproduce the main impairments observed in heart failure condition, such as reduced sensitivity of baroreflex and metabolic controls, lower perfusion to the exercising regions (from 0.6 to 1.4 l/min) and hyperventilation (from 9.2 to 40.2 l/min). The simulator we developed is a useful tool for the description of the basic physiological mechanisms operating during exercise. It can reproduce how these mechanisms interact and how their impairments could limit exercise performance in heart failure condition. The simulator can thus be used in the future as a test bench for different therapeutic strategies aimed at improving exercise performance in cardiopathic subjects.

## Introduction

Physical exercise is associated with an increase in metabolic activity to which the cardiovascular system responds by accommodating a cardiac output eightfold its baseline value, or even higher. Several mechanisms are involved, such as: heart rate increase, heart contractility improvement, higher venous return, vascular vasodilation in the exercising regions, deepening of ventilation pattern (Balady et al., 2010).

In the presence of cardiac pathologies, one or more of these mechanisms are impaired so that patients experience exercise intolerance. Even subjects asymptomatic at rest condition, such as heart failure patients with preserved ejection fraction, show a reduced exercise performance. For this reason the exercise test is nowadays recognized to be a valuable diagnostic tool for the early detection, or the evaluation of a patient's cardiac and pulmonary diseases (Balady et al., 2010). Exercise intolerance in heart failure condition (*HF*) is the result of several physiological impairments involving both central and peripheral mechanisms. *HF* subjects are characterized by a compromised Frank-Starling mechanism, an impaired autonomic and vascular function and a reduced muscular strength (Mezzani et al., 2009). All these factors reduce exercise performance and quality of life in comparison to healthy condition (*Healthy*).

The analysis of these limiting factors and how they fail to fully adapt during exercise can greatly benefit from the use of a dedicated simulator. The simulator has the advantage that it can provide a quantitative description and a rational cause-effect relationship of physiological events. As previously stated, exercise is the result of complex and multifactorial phenomena. Their representation therefore requires a general cardiorespiratory model, combined with its main control mechanisms.

Previous simulators modeled exercise physiology (Heldt et al., 2002; Magosso and Ursino, 2002; Wang et al., 2007) but they did not include the gas exchange in lungs and tissues nor the ventilation control. Cardiovascular-respiratory models have been developed (Batzel et al., 2007; Cheng et al., 2010) but they are not focused on the representation of physical activity phenomena. Finally, the HumMod (Hester et al., 2011), a model of integrative human physiology, provides a representation of the response of the human body to exercise but its structure is quite complex, as it has been developed for several other general applications.

A cardiorespiratory simulator specifically developed to reproduce the basic mechanisms occurring during exercise, and especially their impairments in *HF*, could therefore provide an innovative tool to describe and investigate exercise physiology.

The simulator we present here is a full closed loop cardiorespiratory system. It was developed and used to reproduce cycling activity at different workloads in *Healthy*. The resulting outputs are discussed in this paper and validated with peer-reviewed physiological literature.

In addition, we further adapted the simulator to reproduce the impairments of control mechanisms in *HF* and the resulting limited exercise performance. A comparative analysis between *Healthy* and *HF* exercise is presented, finally, in terms of hemodynamic and respiratory parameters.

## Materials and methods

### General overview of the simulator

**Figure 1:**
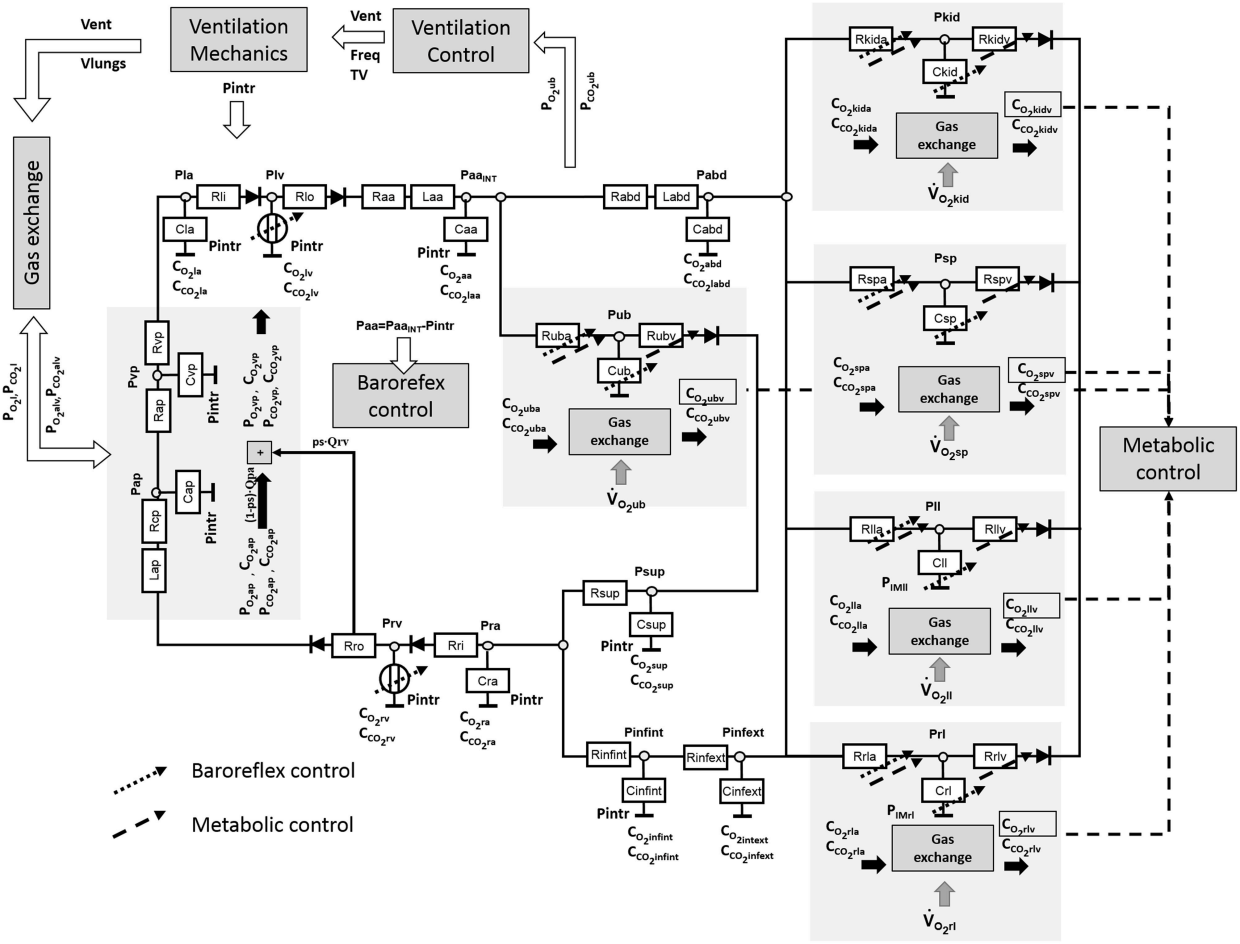
**Block diagram of the cardiorespiratory simulator with all its components**.

**Figure 2:**
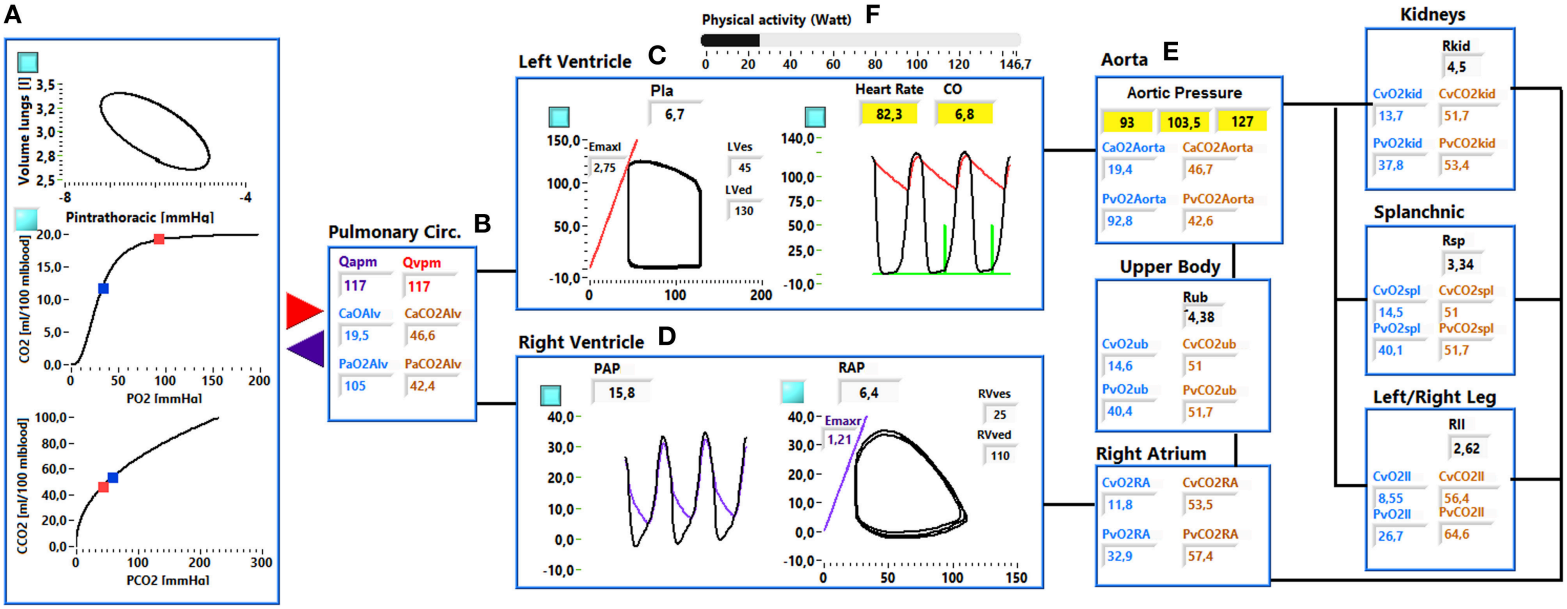
**Simulator interface**. **(A)** Shows: the pressure—volume loop of the ventilation system, the O_2_ and CO_2_ dissociation curve. Red and blue dots indicate the arterial and venous pulmonary concentration and partial pressures values. **(B)** Shows the mean arterial and venous pulmonary flows (*Qapm, Qvpm*) and the *C*_*CO*2_, *C*_*O*2_, *P*_*O*2_, and *P*_*CO*2_ in the alveoli. **(C)** shows the left ventricular pressure—volume loop (left side) and the systemic arterial and left ventricular pressure waveforms (right side). **(D)** Shows the right ventricular and pulmonary arterial pressure waveforms (left side) and the right ventricular pressure-volume loop (right side). **(E)** Shows the arterial resistance (*Ri*), the *C*_*CO*2_, *C*_*O*2_, *P*_*O*2_, and *P*_*CO*2_ in the ith vascular compartment. **(F)** Shows the physical activity regulation button.

The cardiorespiratory simulator is a lumped parameter model developed in LabVIEW 2014 (National Instrument, Austin, TX, USA). The overview of all its components is provided in Figure 1, the interface is shown in Figure 2. Table 1 reports a list of the main abbreviations used in the text.

**Table 1:** **List of abbreviations**.

**Symbol**	**Abbreviation**
*C_*CO*2*ia*_/C_*CO*2*iv*_*	Arterial/venous blood CO_2_ concentration in the ith compartment
*C_*O*2*ia*_/C_*O*2*iv*_*	Arterial/venous blood O_2_ concentration in the ith compartment
*F_*O*2*alv*_/F_*O*2*I*_*	Molar fraction of O_2_ in the alveoli/inspired air
*Fas*	Afferent nerve activity
*Fes*	Sympathetic nerve activity
*Fev*	Vagal nerve activity
*Freq*	Frequency of ventilation
*H*	Generic cardiovascular parameter
*Healthy*	Healthy condition
*HF*	Heart failure condition
*HR*	Heart rate
*P_*CO*2*i*_*	Partial pressure of CO_2_ in the ith compartment
*Pintr*	Intrathoracic pressure
*Pla*	Left atrial pressure
*Plv*	Left ventricular pressure
*Pm*	Mouth pressure
*P_*O*2*i*_*	Partial pressure of O_2_ in the ith compartment
*Ppl*	Pleural pressure
*Qlla*	Left leg arterial blood flow
*Ri*	Arterial or venous resistance of the ith compartment
*Ria*	Arterial resistance of the ith compartment
*Riv*	Venous resistance of the ith compartment
*RQ*	Respiratory quotient
*sf_*Hs*_*	Static function of the sympathetic control for *H*
*sf_*Hv*_*	Static function of the vagal control for *H*
*sf_*RiMet*_*	Static function of the metabolic control for *Ri*
*TC*	Heart cycle duration
*TPR*	Total peripheral resistance
*TV*	Tidal volume
${\overset{•}{V}}_{A}$	Alveolar ventilation
*Vap*	Pulmonary arterial volume
*Vent*	Minute ventilation
*Vlungs*	Lungs volume
*Vla*	Left atrial volume
*Vlv*	Left ventricular volume
${\overset{•}{V}}_{O_{2}i}$	O_2_ consumption in the ith compartment
*WL*	Workload

### Cardiovascular model

The cardiovascular model was already described in Fresiello et al. (2013) and Fresiello et al. (2015). Briefly, atria are represented as passive compliances:

(1) $$\begin{array}{l} Cla=\frac{dVla\left(t\right)}{dPla\left(t\right)} \end{array}$$

Where *Cla* represents the elastic properties of the left atrium, *Vla* and *Pla* are the left atrial volume and pressure, respectively.

Ventricular contraction is described by the time varying elastance model (Sagawa et al., 1988):

(2) $$\begin{array}{l} Plv\left(t\right)=Elv\left(t\right)\cdot \left(Vlv\left(t\right)-Vlv0\right) \end{array}$$

Where *Elv* is the time varying left ventricular elastance with peak systolic value *Elmax, Vlv0* is the left ventricular zero pressure filling volume.

Ventricular filling is represented as a sum of exponential functions:

(3) $$\begin{array}{l} Plv\left(t\right)=al\cdot e^{bl\cdot Vlv\left(t\right)}+cl \end{array}$$

Where *Plv* (*Vlv*) is the left ventricular pressure (volume). The three parameters *a, b* and *c* are estimated to reproduce data from Carroll et al. (1983). Similar equations were implemented for the right atrium and ventricle.

The systemic circulation was already presented in Fresiello et al. (2013). It includes the following sections: ascending aorta, descending aorta, upper body, kidneys, splanchnic circulation, left and right legs, superior vena cava, inferior vena cava inside and outside the chest (see Figure 1). For this latter a Starling resistor was introduced to reproduce the effect of ventilation pressures on the collapsible tube (Pedley, 1980). Venous valves are simulated as simple diodes preventing blood flowing backward. The pulmonary circulation is split into arterial and venous sections (see Ferrari et al., 2011 for more details).

The complete list of cardiovascular parameters is reported in Table 2.

**Table 2:** **List of cardiovascular parameters used for exercise simulation in ***Healthy*** condition**.

**Symbol**	**Parameter**	**Unit**	**Value**	**References**
*HR*	Heart Rate	bpm	58	Ogoh et al., 2005
*Cla/Cra*	Left/Right atrium compliance	cm^3^/mmHg	25/25	Fresiello et al., 2015
*Vlv0/Vrv0*	Left/Right ventricular zero pressure volume	cm^3^	5/5	
*al/ar*	Left/Right ventricular filling	mmHg	0.033/0.05	est. Carroll et al., 1983
*bl/br*		cm^−3^	0.034/0.04	
*cl/cr*		mmHg	8/5	
*Elmax/Ermax*	Left/right ventricular elastance	mmHg/cm^3^	2.5/1.1	est. Sullivan et al., 1989
*Rli/Rri*	Left/Right ventricular input resistance	mmHg·s/cm^3^	0.02	Fresiello et al., 2015
*Rlo/Rro*	Left/Right ventricular output resistance	mmHg·s/cm^3^	0.02	
*Raa*	Ascending aorta/aortic arch resistance	mmHg·s/cm^3^	0.01	
*Laa*	Ascending aorta/aortic arch inertance	mmHg·s^2^/cm^3^	5.10−5	
*Caa*	Ascending aorta/aortic arch compliance	cm^3^/mmHg	0.8	
*Rabd*	Descending aorta resistance	mmHg·s/cm^3^	0.07	
*Labd*	Descending aorta inertance	mmHg·s^2^/cm^3^	5.10−5	
*Cabd*	Descending aorta compliance	cm^3^/mmHg	0.6	
*Ruba_*SET*_*	Upper body arterial resistance	mmHg·s/cm^3^	3.52	est. Sullivan et al., 1989
*Cub*	Upper body compliance	cm^3^/mmHg	8	Heldt et al., 2002
*Rubv_*SET*_*	Upper body venous resistance	mmHg·s/cm^3^	0.23	
*Vub0_*SET*_*	Upper body zero pressure volume	cm^3^	650	
*Rkida_*SET*_*	Kidney arterial resistance	mmHg·s/cm^3^	3.62	est. Sullivan et al., 1989
*Ckid*	Kidney compliance	cm^3^/mmHg	15	Heldt et al., 2002
*Rkidv_*SET*_*	Kidney venous resistance	mmHg·s/cm^3^	0.3	
*Vkid0_*SET*_*	Kidneys body zero pressure volume	cm^3^	150	
*Rspa_*SET*_*	Splanchnic arterial resistance	mmHg·s/cm^3^	2.69	est. Sullivan et al., 1989
*Csp*	Splanchnic compliance	cm^3^/mmHg	55	Heldt et al., 2002
*Rspv_*SET*_*	Splanchnic venous resistance	mmHg·s/cm^3^	0.18	
*Vsp0_*SET*_*	Splanchnic body zero pressure volume	cm^3^	1300	
*Rlla_*SET*_/Rrla_*SET*_*	Left/Right leg arterial resistance	mmHg·s/cm^3^	12.6/12.6	est. Sullivan et al., 1989
*Cll/Crl*	Left/Right leg compliance	cm^3^/mmHg	9.5/9.5	Heldt et al., 2002
*Rllv_*SET*_/Rrlv_*SET*_*	Left/Right leg venous resistance	mmHg·s/cm^3^	0.6/0.6	
*Vll0_*SET*_/Vrl0_*SET*_*	Left/Right leg zero pressure volume	cm^3^	175/175	
*Csup*	Superior vena cava compliance	cm^3^/mmHg	15	
*Rsup*	Superior vena cava resistance	mmHg·s/cm^3^	0.06	
*Cinfext/Cinfint*	Lower vena cava compliance	cm^3^/mmHg	25/2	
*Rinfext/Rinfint*	Lower vena cava resistance	mmHg·s/cm^3^	0.01/0.015	
*Rcp*	Pulmonary characteristic resistance	mmHg·s/cm^3^	0.03	Ferrari et al., 2011
*Cap*	Pulmonary arterial compliance	cm^3^/mmHg	1	
*Rap*	Pulmonary arterial resistance	mmHg·s/cm^3^	0.075	Sullivan et al., 1989
*Lap*	Pulmonary arterial inertance	mmHg·s^2^/cm^3^	3.6.10−5	Ferrari et al., 2011
*Vap0*	Pulmonary arterial zero pressure volume	cm^3^	90	
*Rvp*	Pulmonary venous resistance	mmHg·s/cm^3^	0.005	
*Cvp*	Pulmonary venous compliance	cm^3^/mmHg	5	
*Vvp0*	Pulmonary venous zero pressure volume	cm^3^	580	
*W*	Body weight	Kg	76	Sullivan et al., 1989

*Parameters were taken from literature or estimated (est.) to obtain a good reproduction of literature data*.

### Ventilation mechanics

The mechanics of the lungs' function were replicated through a simplified model taken from Ben-Tal (2006):

(4) $$\begin{array}{l} Pm-Ppl\left(t\right)=R\frac{dVlungs\left(t\right)}{dt}+Vlungs\left(t\right)\cdot E \end{array}$$

Where *R* is the resistive element of the airways, whose value was taken from Ben-Tal (2006). *E* is the elastance element for the lungs whose value was taken from Cross et al. (2012). *Vlungs* is the lungs volume, *Pm* is the mouth pressure set equal to the atmospheric pressure and *Ppl* is the pleural pressure. The latter was reproduced with a sinusoidal function:

(5) $$\begin{array}{l} Ppl\left(t\right)=Ppl_{0}-\frac{E\cdot TV}{2}\cdot \text{sin}\left(\frac{2\cdot \pi \cdot Freq}{60}\cdot t\right) \end{array}$$

Where *Freq* is the ventilation frequency, *TV* is the tidal volume and *Ppl*_0_ is a constant parameter that represents the mean value of *Ppl*. The intrathoracic pressure (*Pintr*) is then calculated as difference between *Ppl* and the atmospheric pressure and is used for all the compliances of the cardiovascular system inside the chest.

### Gas exchange

Gas exchange in the lung compartment is modeled through a mass balance equation (see Appendix in Supplementary Material):

(6) $$\begin{array}{l} ((V_{E\exp}+V_{A}(t))+863\cdot Vap(t)\cdot \frac{dC_{O_{2}ap}(t)}{dP_{O_{2}alv}(t)})\cdot \frac{dP_{O_{2}alv}(t)}{dt}\\ =863\cdot (1-ps)\cdot Qpv(t)\cdot (C_{O_{2}ap}(t)-C_{O_{2}vp}(t))\\ +{\overset{•}{V}}_{A}(t)\cdot (P_{O_{2}I}-P_{O_{2}alv}(t)) \end{array}$$

(7) $$\begin{array}{r} \left(\left(V_{Einsp}+V_{A}\left(t\right)\right)+863\cdot Vap\left(t\right)\cdot \frac{dC_{O_{2}ap}\left(t\right)}{dP_{O_{2}alv}\left(t\right)}\right)\cdot \frac{dP_{O_{2}alv}\left(t\right)}{dt}\\ \;=863\cdot \left(1-ps\right)\cdot Qpv\left(t\right)\cdot \left(C_{O_{2}ap}\left(t\right)-C_{O_{2}vp}\left(t\right)\right) \end{array}$$

Where *Vap* is the pulmonary arterial volume, *V_Eexp_(V_Einsp_)* is the alveolar volume at the end of expiration (inspiration), *V*_*A*_ is the incremental alveolar volume ${\overset{\cdot }{V}}_{A}$ is the alveolar ventilation over time calculated from *dVlungs/dt* in equation (4) subtracting the dead space ventilation calculated from equation (12). *Qpv* is the pulmonary venous blood flow, *ps* is the pulmonary shunt, *C*_*O*2*ap*_ (*C*_*O*2*vp*_) is the O_2_ concentration in the arterial (venous) pulmonary blood, *P*_*O*2*alv*_ (*P*_*O*2*I*_) is the O_2_ partial pressure in the alveoli (inspired air).

We assumed that the blood has enough time to be saturated while flowing in the pulmonary circulation, therefore O_2_ and CO_2_ partial pressures are equal in the alveoli and in the blood. This assumption is valid unless we consider extreme levels of exercise, which is not the aim of the present work.

The O_2_ and CO_2_ concentrations in the blood leaving the lungs are calculated using the dissociation curve developed by Spencer et al. (1979) and Gólczewski (2010), respectively. The O_2_ and CO_2_ concentrations in the arterial blood are calculated combining the concentrations of blood leaving the lungs with the mixed venous blood, according to the *ps* value.

In the tissue compartment, gas exchange is modeled with a mass balance equation:

(8) $$\frac{d\left(C_{O_{2}iv}(t)\cdot Vi(t)\right)}{dt}=C_{O_{2}ia}(t)\cdot Qia(t)-C_{O_{2}iv}(t)\cdot Qiv(t)-{\overset{•}{V}}_{\text{​​}O_{2}i}$$

Where *Qia* (*Qiv*) is the arterial (venous) blood flowing inside (outside) the *ith* compartment, *C*_*O*2*ia*_ is the O_2_ concentration in the arterial blood stream, *C*_*O*2*iv*_ is the O_2_ concentration in the venous blood stream, *Vi* is the blood volume of the *ith* compartment, ${\overset{•}{V}}_{O_{2}i}$ is the O_2_ consumption.

A similar equation was implemented for the CO_2_ with a production term that takes into account the respiratory quotient (*RQ*).

(9) $$\begin{array}{l} \frac{d\left(C_{CO_{2}iv}(t)\cdot Vi(t)\right)}{dt}\\ \;=C_{CO_{2}ia}(t)\cdot Qia(t)-C_{CO_{2}iv}(t)\cdot Qiv(t)+RQ\cdot {\overset{•}{V}}_{O_{2}i} \end{array}$$

We also assume that the diffusion of O_2_ and CO_2_ is fast enough to consider that their concentration in the tissue is equal to the one in the venous blood.

### Ventilation control

Ventilation control takes the arterial partial pressure of O_2_ and CO_2_ in the upper body (*P*_*O*2*uba*_ and *P*_*CO*2*uba*_) as input and provides ventilation (*Vent*) in l/min as output:

(10) $$\begin{array}{l} Vent=\alpha \cdot e^{\beta \cdot P_{O_{2\;uba}}}\cdot \left(P_{{CO}_{2uba}}-P_{CO_{2}tr}\right)+\gamma \cdot \left(P_{{CO}_{2uba}}-P_{CO_{2}tr}\right) \end{array}$$

Equation (10) is an adaptation of the ventilation control developed by Batzel et al. (2007). *P*_*CO*2*tr*_ is a threshold to start a new ventilation cycle, β is a constant parameter, α and γ are the ventilation control gains for O_2_ and CO_2_, respectively. Parameter values were estimated by fitting the data reported by Cormack et al. (1957) and Nunn (1969).

*Vent* is then expressed as frequency (*Freq*) and *TV*:

(11) $$\begin{array}{l} Freq=\delta \cdot Vent+\varepsilon \end{array}$$

Where δ and ε are constant parameters obtained by fitting data from seven healthy subjects reported by Weber et al. (1982). *TV* can be then calculated as the ratio *Vent*∕*Freq* and used in Equation (5).

The effective tidal volume used to calculate alveolar ventilation will be then:

(12) $$\begin{array}{l} TV_{eff}=TV\cdot \left(1-K_{DV}\right) \end{array}$$

Where *K*_*DV*_ is the dead volume ratio that takes into account the percentage of dead volume of airways. The value of this parameter was obtained according to the equation reported by Wasserman et al. (1997): *dead space/tidal volume* = −0.012·*(peak O*_2_ *uptake)* + 0.611. We considered a peak O_2_ uptake of 34 and 15 ml/min/Kg for *Healthy* and *HF*, respectively.

A list of ventilation parameters is reported in Table 3.

**Table 3:** **List of parameters used for the ventilation and the muscle contraction models**.

**Symbol**		**Equations**	**Unit**	**Value (Healthy)**	**Value (HF)**	**References**
*K_*DV*_*	Dead volume ratio	(12)		0.8	0.57	Wasserman et al., 1997
*P_*CO*2*tr*_*	P_*CO*2_ threshold for ventilation onset	(10)	mmHg	36.75		Batzel et al., 2007
*E*	Lungs elastance	(4)	mmHg/l	2.0	2.8	Cross et al., 2012
*P_*CO*2*I*_*	P_*CO*2_ in the inflow air	(7)	mmHg	0		
*P_*IMmax*_*	Peak value of *P_*IM*_* per unit of *WL*	(29)	mmHg/W	0.562		est. Rådegran and Saltin, 1998
*Ppl_0_*	Mean value of *Ppl*	(5)	mmHg	754		Ben-Tal, 2006
*P_*O*2*I*_*	P_*O*2_ in the inflow air	(6)	mmHg	150		
*ps*	Pulmonary shunt ratio	(6)–(7)		0.02		Whiteley et al., 2003
*R*	Airways resistances	(4)	mmHg/(l/s)	1		Ben-Tal, 2006
α	Control gain of ventilation for O_2_	(10)	l/(min·mmHg)	30		est. Cormack et al., 1957; Nunn, 1969
β			mmHg^−1^	−0.055		
γ	Control gain of ventilation for CO_2_		l/(min·mmHg)	2		
δ	*Freq* to *Vent* relationship parameters	(11)	min/l	0.274		est. Weber et al., 1982
ε				17.75		

*Parameters were taken from literature or estimated (est.) to obtain a good reproduction of literature data*.

### Baroreflex control

The baroreflex model was taken from Ursino (1998) and Fresiello et al. (2013). It provides a representation of the afferent nerve activity, depending on the pressure sensed in the aortic region. In addition, the model reproduces the vagal and sympathetic nerve activity and their effects on cardiovascular parameters. In the model of Ursino (1998) the pressure in the carotid arteries is the input the baroreflex control. Since in the present simulator there is no specific representation of the carotid arteries, the aortic pressure without the effect of the intrathoracic pressure was considered as input pressure for the baroreflex control (*Paa*). This pressure is used in a linear derivative block:

(13) $$\begin{array}{l} \tau_{p}\cdot \frac{dP\left(t\right)}{dt}=Paa\left(t\right)+\tau_{z}\cdot \frac{dPaa\left(t\right)}{dt}-P\left(t\right) \end{array}$$

Where τ_*p*_ and τ_*z*_ are the pole and the real zero. The output variable *P(t)* has the dimension of a pressure.

To reproduce exercise and the related phenomenon of baroreflex resetting, the model was further changed. Three main mechanisms were implemented: the change of systemic arterial pressure set point *Paa*_*SET*_ (strictly related to the operating point of baroreflex), the progressive increment of sympathetic activity (*Fes*), and the vagal (*Fev*) withdrawal.

The change of *Paa*_*SET*_ was modeled as a function of workload level (*WL*):

(14) $$\begin{array}{l} Paa_{SET}=Paa_{SET0}+A\cdot WL \end{array}$$

Where *Paa*_*SET*0_ is the set-point pressure at rest condition and *A* is the rate of increase of *Paa*_*SET*_ per workload unit. Its value was estimated to reproduce the data reported by Ogoh et al. (2005).

*Paa*_*SET*0_ is used for the calculation of the afferent sympathetic activity *Fas*:

(15) $$\begin{array}{l} Fas\left(t\right)=\frac{Fas_{\text{min}}+Fas_{\text{max}}\cdot e^{\left(\frac{P\left(t\right)-Paa_{SET}}{ka}\right)}}{1+e^{\left(\frac{P\left(t\right)-Paa_{SET}}{ka}\right)}} \end{array}$$

*Fas*_*max*_ and *Fas*_*min*_ are constant parameters representing the upper and lower saturation levels of the *Fas* function, *ka* is a constant parameter related to the slope of *Fas* at the central point (obtained for *P(t)* = *Paa*_*SET*_).

*Fas* is used to compute the sympathetic nerve activity (*Fes*):

(16) $$\begin{array}{l} Fes\left(t\right)=Fes_{\infty }+\left(Fes_{0}-Fes_{\infty }\right)\cdot e^{-kes\cdot Fas\left(t\right)}+\Delta_{Fes} \end{array}$$

Where *Fes*_0_, *Fes*_∞_ and *kes* are constant parameters, Δ_*Fes*_ is the progressive sympathetic stimulation due to exercise onset. It was implemented as a function of *WL*:

(17) $$\begin{array}{l} \Delta_{Fes}=B\cdot WL \end{array}$$

Where *B* is the rate of *Fes* increase per workload unit. *Fas* is also used to compute the efferent vagal activity (*Fev*):

(18) $$\begin{array}{l} Fev\left(t\right)=\frac{Fev_{0}+Fev_{\infty }\cdot e^{\left(\frac{Fas\left(t\right)-Fas_{0}}{kev}\right)}}{1+e^{\left(\frac{Fas\left(t\right)-Fas_{0}}{kev}\right)}}+\Delta_{Fev} \end{array}$$

Where *Fev*_∞_ is the lower limit of vagal nerve activity, *kev* is a constant parameter related to the slope of the function at the central point (obtained for *P(t)* = *Paa*_*SET*_). Δ*fev* represents the vagal nerve activity withdrawal and is expressed as a function of *WL*:

(19) $$\begin{array}{l} \Delta_{Fev}=C\cdot WL \end{array}$$

Where *C* is the rate of *Fev* increase per workload unit.

*Fev*_∞_ in Equation (19) is the upper limit of vagal nerve activity and is also expressed as a function of *WL*:

(20) $$\begin{array}{l} Fev_{\infty }=Fev_{\infty 0}+D\cdot WL \end{array}$$

Where *Fev*_∞0_ is the upper limit of *Fev* at rest, *D* is the rate of decrease of *Fev*_∞_ so to assure that at intensive exercise levels, *Fev* = 0, even at higher pressure levels.

The parameters in Equations (17), (19), and (20) were estimated according to the data of Robinson et al. (1966) relative to the sympathetic and parasympathetic controls of *HR* in humans during exercise. In addition, to estimate the parameters in Equations (19) and (20), we also imposed a nearly complete vagal withdrawal when *HR* reaches 100 bpm during exercise. This is in agreement with what was reported by Rowell and O'Leary (1990).

*Fes* and *Fev* are then used to obtain the static sympathetic and vagal functions (*sf*_*Hs*_ and *sf*_*Hv*_):

(21) $$\begin{array}{l} sf_{Hs}\left(t\right)=C_{Hs}\cdot \left(\text{ln }\left(Fes\left(t-D_{Hs}\right)-Fes_{SET}-1.65\right)-1.1\right) \end{array}$$

(22) $$\begin{array}{l} sf_{Hv}\left(t\right)=C_{Hv}\cdot \left(Fev\left(t-D_{Hv}\right)-Fev_{SET}\right) \end{array}$$

Where *D*_*Hs*_ (*D*_*Hv*_) is the sympathetic (vagal) delay, *C*_*Hs*_ (*C*_*Hv*_) is the sympathetic (vagal) control gain for the parameter *H*. *Fes*_*SET*_ (*Fev*_*SET*_) is the value of *Fes* (*Fev*) at the central point (obtained for *Paa* = *Paa*_*SET*_).

The final control of the cardiovascular parameter *H* due to sympathetic nerve activity will be:

(23) $$\begin{array}{r} \frac{d\Delta H_{s}\left(t\right)}{dt}=\frac{sf_{Hs}\left(t\right)-\Delta H_{S}}{T_{Hs}}\\ H\left(t\right)=H_{SET}+\Delta H_{s}\left(t\right) \end{array}$$

Where Δ*H*_*s*_ is the change of *H* due to the sympathetic control, *H*_*SET*_ is the set-point value of *H*, and *T*_*Hs*_ is the time constant of the sympathetic control. For the vagal control a similar equation was implemented.

The model is arranged in such a way that for *Paa* = *Paa*_*SET*_ the hemodynamic parameters assume their set-point value (*H* = *H*_*SET*_). If *Paa* differs from *Paa*_*SET*_ the baroreflex will induce a change of the hemodynamic parameters. In particular the sympathetic control will affect the left and right ventricular contractility, the arterial resistance and the venous tone. For *HR* both sympathetic and parasympathetic controls are considered so that the final regulation will be:

(24) $$\begin{array}{l} \frac{d\Delta TC_{s}(t)}{dt}=\frac{sf_{TCs}(t)-\Delta TC_{s}}{T_{TCs}}\\ \frac{d\Delta TC_{v}(t)}{dt}=\frac{sf_{TCv}(t)-\Delta TC_{v}}{T_{TCv}}\\ \;TC(t)=TC_{SET}+\Delta TC_{s}(t)+\Delta TC_{v}(t) \end{array}$$

Where *TC* is duration of a cardiac cycle, *TC*_*SET*_ is the set-point *TC*, Δ*TC*_*s*_, and Δ*TC*_*v*_ are the changes due to sympathetic and vagal nerve activity, respectively.

A list of parameters used for baroreflex resetting and control is reported in Tables 4, 5.

**Table 4:** **List of parameters used for baroreflex model**.

**Symbol**		**Equations**	**Unit**	**Value (*Healthy*)**	**Value (*HF*)**	**References**
A	Rate of Paa_*SET*_ increase per unit of *WL*	(14)	mmHg/W	0.242	0.3517	est. Ogoh et al., 2005
*B*	Rate of *Fes* increase per unit of *WL*	(17)	spike/(W·s)	0.12	0.02	
*C*	Rate of *Fev* decrease per unit of *WL*	(19)	spike/(W·s)	−0.041		est. Robinson et al., 1966
*D*	Rate of *Fev*_8_decrease per unit of *WL*	(20)	spike/(W·s)	−0.044		est. Robinson et al., 1966
*ka*	*Fas* slope parameter	(15)	mmHg	11.758		Ursino, 1998
*kes*	*Fes* slope parameter	(16)	s	0.0675		
*kev*	*Fev* slope parameter	(18)	spikes/s	7.06		
*Fes*_0_	*Fes* upper limit	(16)	spikes/s	16.11		
*Fes*_∞_	*Fes* lower limit	(16)	spikes/s	2.10		
*Fev*_0_	*Fev* lower limit	(18)	spike/s	3.2		
*Fev*_∞0_	*Fev* upper limit at rest	(20)	spike/s	6.3		
Paa_*SET*0_	Set point pressure	(14)	mmHg	90	93	Sullivan et al., 1989
τ_*p*_	Pole	(13)	s	2.076		Ursino, 1998
τ_*z*_	Zero	(13)	s	6.37		

*Parameters were taken from literature or estimated (est.) to obtain a good reproduction of literature data*.

**Table 5:** **List of parameters used for the sympathetic (***symp***), vagal (***vag***), and metabolic (***met***) controls**.

**Symbol**		**Equations**	**Unit**	**Value (*Healthy*)**	**Value (*HF)***	**References**
*C_*TCs*_*	*TC* symp control gain	(21)	s/(spikes/s)	−0.09	−0.0594	Ursino, 1998 (*Healthy*)
*C_*TCv*_*	*TC* vag control gain	(22)	s/(spikes/s)	0.07	0.0462	est. Ogoh et al., 2005 (*HF*)
*C_*Elmaxs*_*	*Elmax* symp control gain	(21)	(mmHg/cm^3^)/(spikes/s)	0.61	0.2	est. Fresiello et al., 2014
*C_*Ermaxs*_*	*Ermax* symp control gain	(21)	(mmHg/cm^3^)/(spikes/s)	0.133	0.133	
*C_*Rubas*_*	*Ruba* symp control gain	(21)	(mmHg·s/cm^3^)/(spikes/s)	1.16	1.62	
*C_*Rkidas*_*	*Rkida* symp control gain	(21)	(mmHg·s/cm^3^)/(spikes/s)	1.10	1.53	
*C_*Rspas*_*	*Rspa* symp control gain	(21)	(mmHg·s/cm^3^)/(spikes/s)	0.95	1.32	
*C_*Rllas*_*	*Rlla* symp control gain	(21)	(mmHg·s/cm^3^)/(spikes/s)	2.4	4.06	
*C_*Rrlas*_*	*Rlra* symp control gain	(21)	(mmHg·s/cm^3^)/(spikes/s)	2.4	4.06	
*C_*RubaMet*_*	*Ruba* met control gain	(26)	mmHg·s/cm^3^	0.73		est. Pawelczyk et al., 1992; Calbet, 2006; Heinonen et al., 2013
*C_*RkidaMet*_*	*Rkida* met control gain	(26)	mmHg·s/cm^3^	0.69		
*C_*RspaMet*_*	*Rspa* met control gain	(26)	mmHg·s/cm^3^	0.6		
*C_*RllaMet*_*	*Rlla* met control gain	(26)	mmHg·s/cm^3^	1.5		
*C_*RrlaMet*_*	*Rlra* met control gain	(26)	mmHg·s/cm^3^	1.5		
*C_*RubvMet*_*	*Rubv* met control gain	(26)	mmHg·s/cm^3^	0.046		
*C_*RkidvMet*_*	*Rkidv* met control gain	(26)	mmHg·s/cm^3^	0.06		
*C_*RspvMet*_*	*Rspv* met control gain	(26)	mmHg·s/cm^3^	0.036		
*C_*RllvMet*_*	*Rllv* met control gain	(26)	mmHg·s/cm^3^	0.12		
*C_*RrlvMet*_*	*Rlrv* met control gain	(26)	mmHg·s/cm^3^	0.12		
*C_*Vub*0*s*_*	*Vub0* symp control gain	(21)	cm^3^/(spikes/s)	−28.1	−28.1	est. Fresiello et al., 2014
*C_*Vkid*0*s*_*	*Vkid0* symp control gain	(21)	cm^3^/(spikes/s)	−6.5	−6.1	
*C_*Vsp*0*s*_*	*Vsp0* symp control gain	(21)	cm^3^/(spikes/s)	−228.3v	−228.3	
*C_*Vll*0_*	*Vll0* symp control gain	(21)	cm^3^/(spikes/s)	−7.8	−7.8	
*C_*Vrl*0_*	*Vlr0* symp control gain	(21)	cm^3^/(spikes/s)	−7.8	−7.8	
*C_*O*2*ubvRef*_*	Reference value for *C_*O*2*ubv*_*	(25)	ml O_2_/dl blood	14	12	Healthy: Lanzarone et al., 2007 HF: est. Sullivan et al., 1989
*C_*O*2*kidvRef*_*	Reference value for *C_*O*2*kidv*_*	(25)	ml O_2_/dl blood	17.5	15.5	
*C_*O*2*spvRef*_*	Reference value for *C_*O*2*spv*_*	(25)	ml O_2_/dl blood	15	13	
*C_*O*2*llvRef*_*	Reference value for *C_*O*2*llv*_*	(25)	ml O_2_/dl blood	14	12	
*C_*O*2*rlvRef*_*	Reference value for *C_*O*2*lrv*_*	(25)	ml O_2_/dl blood	14	12	
*k_*MET*_*	*sf_*RiMet*_* slope parameter	(25)	dl blood/ml O_2_	1.8		est. Pawelczyk et al., 1992; Calbet, 2006; Heinonen et al., 2013
*S_0_*	Ratio of basal arterial resistance	(28)		0.27		
*T_*MET*_*	Time constant met control	(26)	s	2		Lanzarone et al., 2007
*T_*Elmax*_*	Time constant *Elmax* symp control	(23)	s	8		Ursino, 1998
*T_*Ermax*_*	Time constant *Ermax* symp control	(23)	s	8		
*T_*Ris*_*	Time constant *Ri* symp control	(23)	s	6		
*T_*TCs*_*	Time constant *TC* symp control	(23)	s	2		
*T_*TCv*_*	Time constant *TC* vag control	(23)	s	1.5		
*T_*Vis*_*	Time constant *Vi* symp control	(23)	s	20		

### Peripheral metabolic control

The metabolic control is a sigmoidal function estimated on the basis of data observed in human subjects (Pawelczyk et al., 1992; Calbet, 2006; Heinonen et al., 2013).

(25) $$\begin{array}{l} sf_{RiMet}\left(t\right)=1-\frac{1}{1+e^{k_{MET}\cdot \left(C_{O_{2i}v}\left(t\right)-\frac{C_{O_{2}ivRef}}{2}\right)}} \end{array}$$

Where *C*_*O*2*iv*_ is the venous oxygen concentration in the i*th* circulatory district and *C*_*O*2*ivRef*_ is its reference value taken from Lanzarone et al. (2007). The static function *sf*_*RiMet*_ is then used in the first order dynamic block that controls the peripheral arterial and venous resistance of each circulatory district:

(26) $$\begin{array}{l} \frac{d\Delta Ri_{Met}\left(t\right)}{dt}=\frac{C_{RiMet}\cdot \left(sf_{RiMet}\left(t\right)-1\right)-\Delta Ri_{Met}\left(t\right)}{T_{Met}} \end{array}$$

Where Δ*Ri*_*Met*_ is the change induced by the metabolic control, *T*_*Met*_ is the time constant of the metabolic control. *C*_*RiMet*_ is the control gain estimated from the data reported by Pawelczyk et al. (1992) and Gonzalez-Alonso et al. (2002). The final control of the venous resistance of the ith vascular compartment (*Riv*) will be:

(27) $$\begin{array}{l} Riv\left(t\right)=Riv_{SET}+\Delta Riv_{Met}\left(t\right) \end{array}$$

Where *Riv*_*SET*_ is the set-point value of the venous resistance of i*th* vascular district. The metabolic control is arranged in a way that if *C*_*O*2*iv*_ = *C*_*O*2*ivRef*_ then *Riv* = *R*_*ivSET*_.

The control of the arterial resistances is discussed in the next paragraph. The list of metabolic control parameters is reported in Table 5.

### Metabolic and baroreflex interaction

An important mechanism during exercise is the sympatholysis which determines the mutual interaction of baroreflex and metabolic systems in the control of peripheral circulation. The metabolic control counteracts sympathetic vasoconstriction in exercising regions, as some local factors and substances reduce the sensitivity of vascular smooth muscle to sympathetic tone (Laughlin et al., 2011). To reproduce the sympatholysis effect we implemented the control of the arterial peripheral resistance as follows:

(28) $$\begin{array}{l} Ria\left(t\right)=Ria_{SET}\cdot S_{0}+\left[Ria_{SET}\cdot \left(1-S_{0}\right)+\Delta Ria_{s}\left(t\right)\right]\cdot \\ sf_{RiMet}^{10}\left(t\right)+\Delta Ria_{MET} \end{array}$$

In Equation (28) the metabolic control *sf*_*RiMet*_(t) affects Δ*Ria*_*s*_*(t)* so that when the metabolic vasodilation occurs, the sympathetic effect also reduces. *S*_0_ is a constant parameter that reproduces the arterial resistance when the sympathetic vasoconstriction is completely abolished (Pawelczyk et al., 1992; Calbet, 2006; Heinonen et al., 2013). Its use is discussed in more detail in paragraph 3.3.1.

### Muscle contraction

Muscle contraction in the exercising regions is represented by a sinusoidal function. We adapted the one reported by Magosso and Ursino (2002) to reproduce different levels of *WL*.

(29) $$\begin{array}{l} \begin{array}{c} P_{IMll}\left(t\right)=P_{IM\text{max}}\cdot WL\cdot \left(1+\text{sin}\left(2\pi \cdot t\right)\right)\\ P_{IMrl}\left(t\right)=P_{IM\text{max}}\cdot WL\cdot \left(1+\text{sin}\left(2\pi \cdot t+\pi \right)\right) \end{array} \end{array}$$

*P*_*IMll*_ and *P*_*IMrl*_ are two sinusoidal functions reproducing the intramuscular pressure of the left and right leg respectively. Their frequency was set to 1 Hz considering a cycling rate of 60 rotations per minute. Their amplitude depends on the value *P*_*IMmax*_ and on the workload set on the bicycle. *P*_*IMmax*_ was estimated on the basis of data reported by Rådegran and Saltin (1998).

### Parameter assignment

Parameter assignment was performed to characterize the simulator at rest condition for *Healthy* and *HF*. Then, the exercise was simulated in both conditions and model output was compared with the data in the literature (see next paragraph).

Cardiovascular parameters were set as reported in Fresiello et al. (2015). Some parameters were taken from Sullivan et al. (1989) referring to average data of 12 healthy subjects and of 30 chronic heart failure patients at rest condition, before starting the exercise test. In particular we set pulmonary resistances, lower limbs' and total systemic arterial resistance and the blood volume on the basis of body weight. A complete list of cardiovascular parameters for *Healthy* at rest is reported in Table 2.

To reproduce *HF* condition we changed the left ventricular systolic and diastolic functions. The choice of parameter values was already explained in Fresiello et al. (2015) and will be omitted here for brevity. Vascular parameters were changed according to data reported by Sullivan et al. (1989). The complete list of cardiac and vascular parameters that were changed for *HF* representation is reported in Table 6.

**Table 6:** **List of cardiovascular parameters used for exercise simulation in ***HF*** condition**.

**Symbol**	**Unit**	**Value**	**References**
*HR*	bpm	85	Sullivan et al., 1989
*Elmax*	mmHg/cm^3^	1.5	Fresiello et al., 2015
*Vlv0*	cm^3^	25	
*al*	mmHg	0.031	
*bl*	cm^−3^	0.031	
*cl*	mmHg	8	
*Rub_*SET*_*	mmHg·s/cm^3^	4.72	Sullivan et al., 1989
*Rkida_*SET*_*	mmHg·s/cm^3^	4.88	
*Rspa_*SET*_*	mmHg·s/cm^3^	3.62	
*Rlla_*SET*_*	mmHg·s/cm^3^	8.52	
*Rrla_*SET*_*	mmHg·s/cm^3^	8.52	
*Rap*	mmHg·s/cm^3^	0.175	

*Parameters were taken from literature or estimated (est.) to obtain a good reproduction of literature data*.

Baroreflex sub-model parameters are reported in Tables 4, 5. Briefly, gain values of the baroreflex control were characterized as reported in Fresiello et al. (2013). The shift of *Paa*_*SET*_ was reproduced according to the data reported by Ogoh et al. (2005). Vagal withdrawal parameters were estimated to reproduce data from Rowell and O'Leary (1990) and Robinson et al. (1966). The sympathetic stimulation parameters were estimated in order to reproduce the data reported by Robinson et al. (1966).

For HF condition the sympathetic stimulation parameters were obtained fitting the data from Sullivan et al. (1989).

The metabolic control function was set so as to obtain a good reproduction of the data in the literature (Pawelczyk et al., 1992; Calbet, 2006; Heinonen et al., 2013). These data refer to the mere metabolic control of peripheral resistance during exercise in the absence of a sympathetic effect. *C*_*O*2*ivRef*_ in *Healthy* was set according to Lanzarone et al. (2007). For the *HF* we considered a lower *C*_*O*2*ivRef*_ at rest, as reported in Sullivan et al. (1989). The complete list of metabolic parameters is provided in Table 5. The sympatholysis function described in Equation (28) was estimated on the basis of data from Pawelczyk et al. (1992) and Gonzalez-Alonso et al. (2002) referring to both sympathetic and metabolic control during exercise.

Ventilation control parameters were obtained by fitting data from Cormack et al. (1957) and Nunn (1969). Parameters relative to ventilation frequency and tidal volume in Equation (11) were estimated by fitting data from Weber et al. (1982).

### Validation procedure

After the assignment of parameters at rest, we simulated graded bicycle exercise from rest to 73 watts. To reproduce *Healthy* exercise we fed the simulator with increasing levels of oxygen consumption:

(30) $${\overset{•}{V}}_{O_{2}RR}=196$$

(31) $${\overset{•}{V}}_{O_{2}ll}={\overset{•}{V}}_{O_{2}rl}=5.87\;\cdot \;WL+20.23$$

Where ${\overset{•}{V}}_{O_{2}RR}$ is resting regions O_2_ uptake, ${\overset{•}{V}}_{O_{2}ll}$ (${\overset{•}{V}}_{O_{2}rl}$) is left (right) leg O_2_ uptake expressed as function of workloads. *V*_*O*2*RR*_ is divided among all resting circulatory branches as follows: 30% for the upper body, 32% for kidney and 38% for splanchnic circulation.

For the *RQ* we used the following formula:

(32) $$\begin{array}{l} RQ=0.0014\cdot WL+0.859 \end{array}$$

A similar procedure was performed for ${\overset{•}{V}}_{O_{2}RR}$, ${\overset{•}{V}}_{O_{2}ll}$ (${\overset{•}{V}}_{O_{2}rl}$) and *RQ* in *HF*:

(33) $${\overset{•}{V}}_{\text{​}O_{2}RR\_HF}=2.76\;\cdot \text{ WL}+201.06$$

(34) $${\overset{•}{V}}_{O_{2}ll\_HF}\text{​}={\overset{•}{V}}_{O_{2}rl\_HF}=3.83\;\cdot \text{ WL}+28.87$$

(35) $$\begin{array}{l} RQ_{\_HF}=0.006\cdot WL+0.877 \end{array}$$

*HF* is characterized by lower values of oxygen uptake in both exercising and resting regions and by an earlier anaerobic metabolism in comparison to *Healthy*.

Equations (30) to (35) were obtained by interpolating data from Sullivan et al. (1989).

To reproduce the exercise we initially set the simulator at rest condition and we left the simulator free to evolve and reach the steady condition at 24.5 watts, 49 watts and 73 watts. For each exercise step data were then averaged over 15 heart cycles and reported as mean values. Simulations were then compared to the exercise test data from Sullivan et al. (1989) concerning isokinetic cycling with a graded workload of +24.5 watts/3 min.

## Results

### Sub-models

This first part of the results' section is devoted to further illustrating some of the sub-models described in the methods section. We focus on baroreflex, metabolic and ventilation controls.

**Figure 3:**
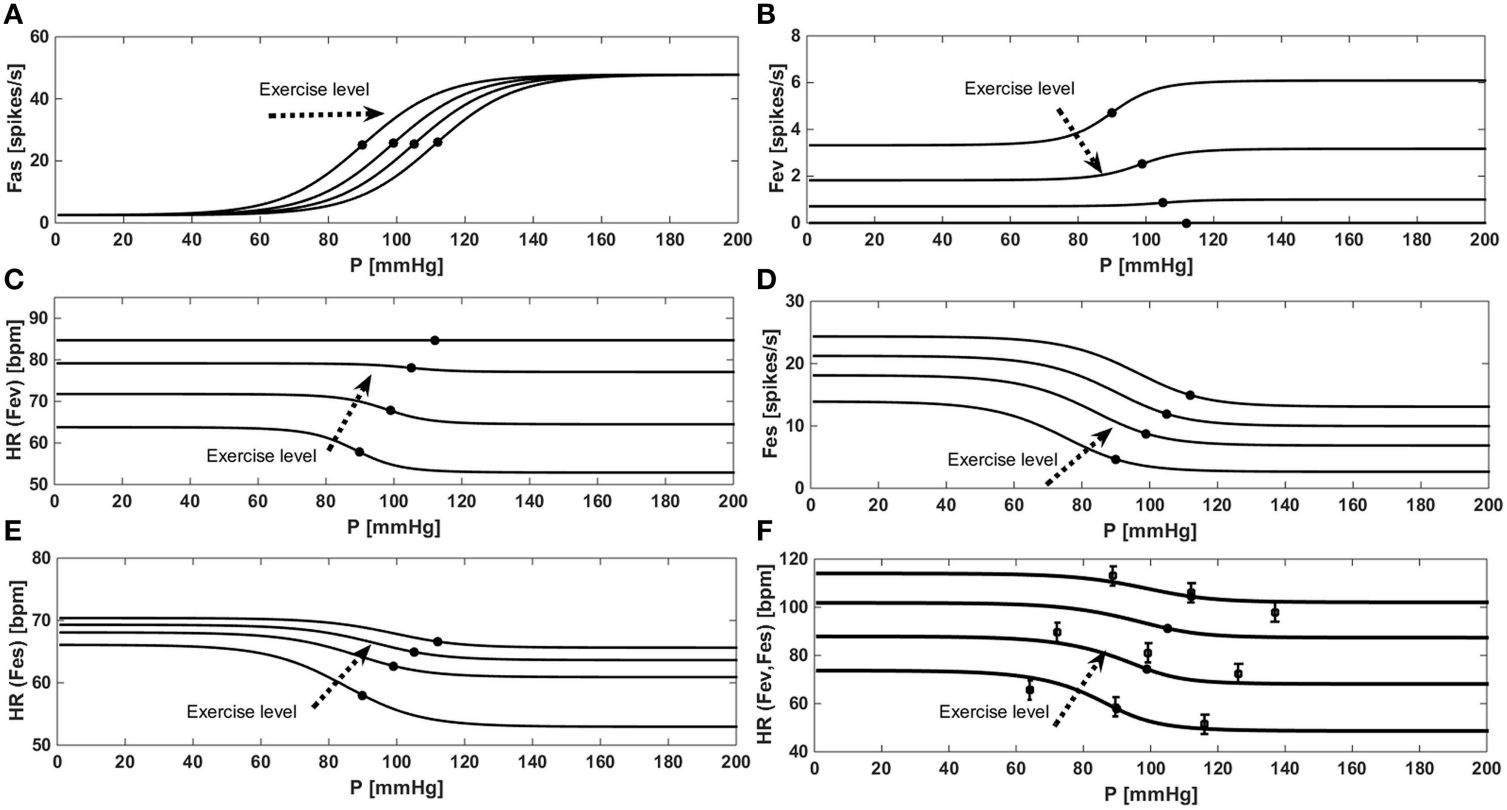
**Results of the baroreflex resetting model for different levels of physical activity**. Dots represent baroreflex central point (for *Paa* = *Paa*_*SET*_). **(A)** *Fas* as a function of aortic pressure in a baroreflex open loop configuration. **(B)** Progressive vagal withdrawal for increasing levels of exercise. **(C)** Effects of vagal withdrawal on *HR*. **(D)** Progressive sympathetic stimulation for increasing levels of exercise. **(E)** Effects of sympathetic stimulation on *HR*. **(F)** Overall effects of baroreflex resetting (both *Fev* and *Fes*) on *HR*, comparison between simulations data (continuous line) and the data (■) from Ogoh et al. (2005).

Figure 3 shows the “baroreflex resetting” described in Equations (13) to (22). We simulated the stimulus-response curve of the baroreflex model in an open-loop configuration, by imposing an aortic pressure ranging from 0 to 200 mmHg. We repeated this procedure at rest condition at three different exercise levels (35, 61, and 87 watts). Figure 3A shows the progressive increase of *Paa*_*SET*_ described in Equation (14) and the relative effects on *Fas* as described in Equation (15). Figure 3B shows the progressive vagal withdrawal with the increasing of the exercise level described in Equations (18)−(20). The effect of vagal withdrawal on *HR* is shown in Figure 3C. We obtained an increment of *HR* of +28 bpm (from 57 to 85 bpm), similar to the average increase of +36 bpm reported by Robinson et al. (1966). Figure 3D shows the sympathetic stimulation for increasing levels of exercise as described in Equations (16) and (17). The related effects on HR are shown in Figure 3C. We obtained an average *HR* increase of +18 bpm (from 58 to 66 bpm), similar to the increase of +16 bpm reported by Robinson et al. (1966). The final control of *HR*, obtained by combining both *Fes* and *Fev*, is shown in Figure 3F. Model results are compared with the data in the literature from Ogoh et al. (2005) relative to rest, 31 watts and 85 watts conditions.

**Figure 4:**
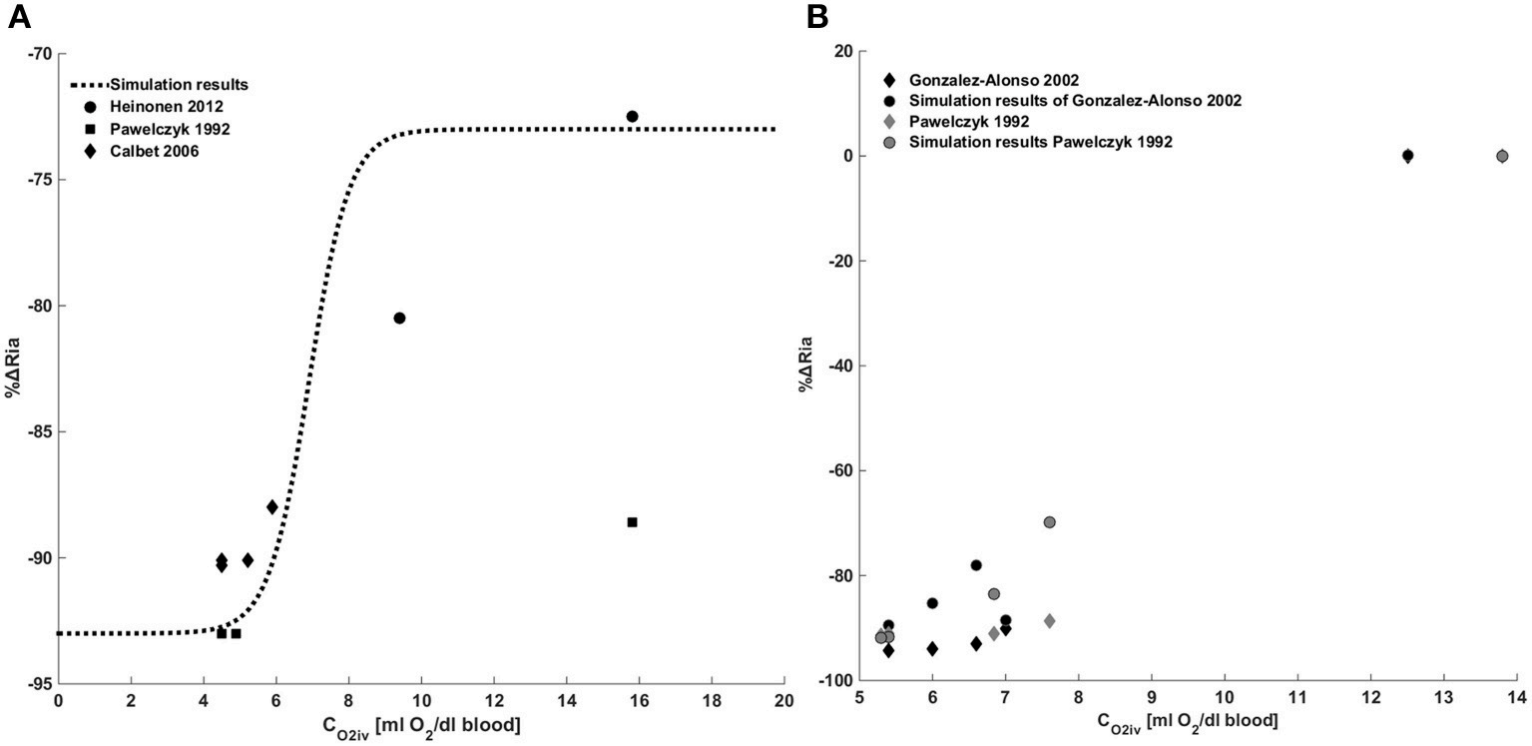
**Metabolic control of peripheral resistance**. **(A)** Percentage change of the peripheral resistance due to a change in venous oxygen concentration during exercise in the absence of a sympathetic control. Comparison between simulations output and the data from Pawelczyk et al. (1992) and Gonzalez-Alonso et al. (2002). **(B)** Percentage change of the peripheral resistance due to a change in venous oxygen concentration during exercise in presence of sympathetic control. Comparison between simulations output and the data from Pawelczyk et al. (1992), Calbet (2006) and Heinonen et al. (2013).

Figure 4 provides a comparison between model results and the data in the literature for the metabolic control. Figure 4A shows a comparison between the model we implemented and the data of Pawelczyk et al. (1992), Calbet (2006) and Heinonen et al. (2013). These data refer to the metabolic control during exercise with a complete suppression of sympathetic vasoconstriction. To reproduce these data we removed the sympathetic contribution to peripheral resistance in Equation (28), obtaining *Ria*(*t*) = *Ria*_*SET*_·*S*_0_ + Δ*Ria*_*MET*_.

Figure 4B shows a comparison between simulation results and the data in the literature taken from Pawelczyk et al. (1992) and Gonzalez-Alonso et al. (2002). These data refer to the systemic resistance regulation during exercise in the presence of both metabolic and sympathetic controls. Results show that for *C*_*O*2*iv*_ = *C*_*O*2*ivRef*_ no changes of resistances are observed, for *C*_*O*2*iv*_<*C*_*O*2*ivref*_ a vasodilation is induced by the metabolic control.

**Figure 5:**
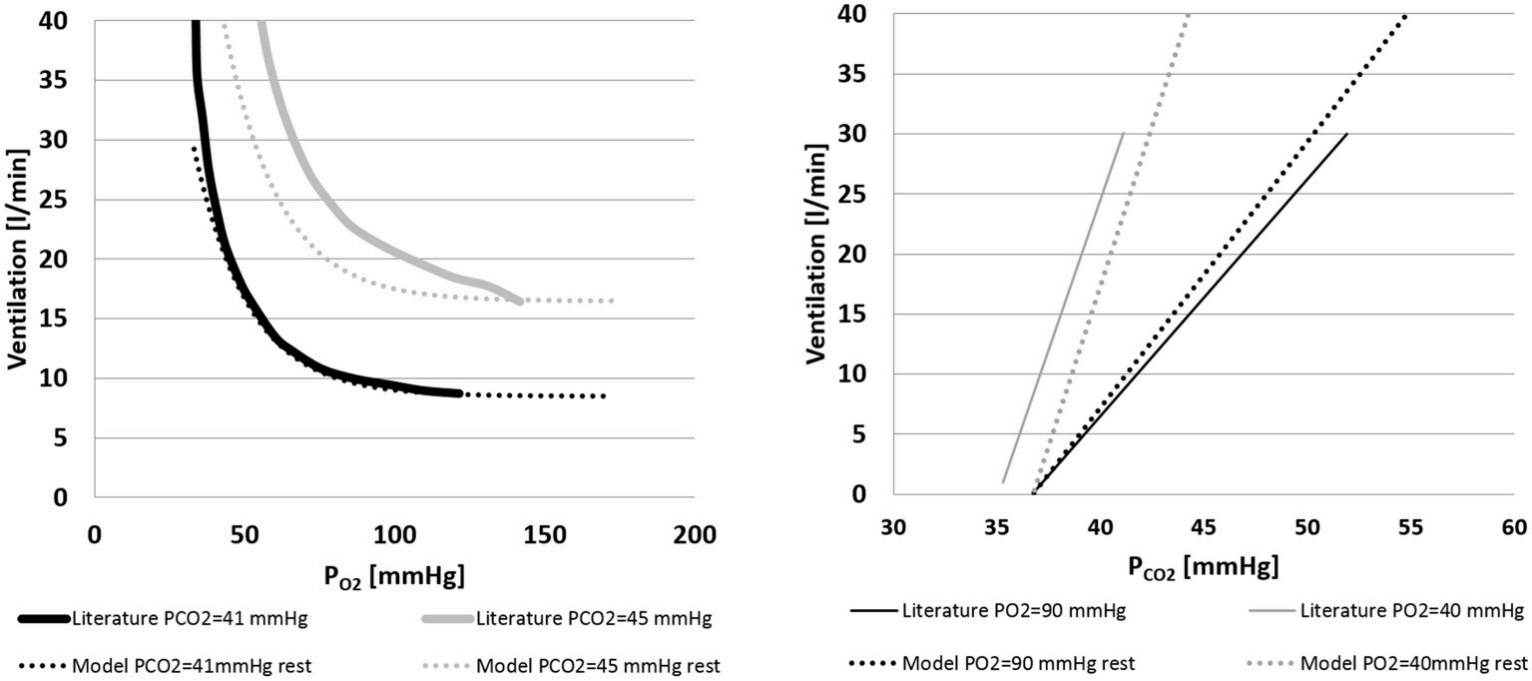
**Left panel:** ventilation over *PO*_2_ for two constant values of *PCO*_2_(41 and 45 mmHg). Comparison between literature (Cormack et al., 1957) and model data. **Right panel:** ventilation over *PCO*_2_ for two constant values of *PO*_2_(40 and 90 mmHg). Comparison between model output and the data from Nunn (1969) and model output.

Figure 5 shows the ventilation control as implemented in Equation (10). Figure 5A shows the ventilation as a function of P_*O*2_ for two constant values of P_*CO*2_. Simulations were repeated at rest condition and at *WL* = 73 watts and compared with data from Cormack et al. (1957). Figure 5B shows the ventilation as a function of P_*CO*2_ for two constant values of P_*O*2_. In this case also, simulations were repeated at rest condition and at *WL* = 73 watts, and results were compared with data from Nunn (1969).

### Exercise data

In this second part of the results' section the output of the cardiovascular simulator for graded exercise is shown.

**Figure 6:**
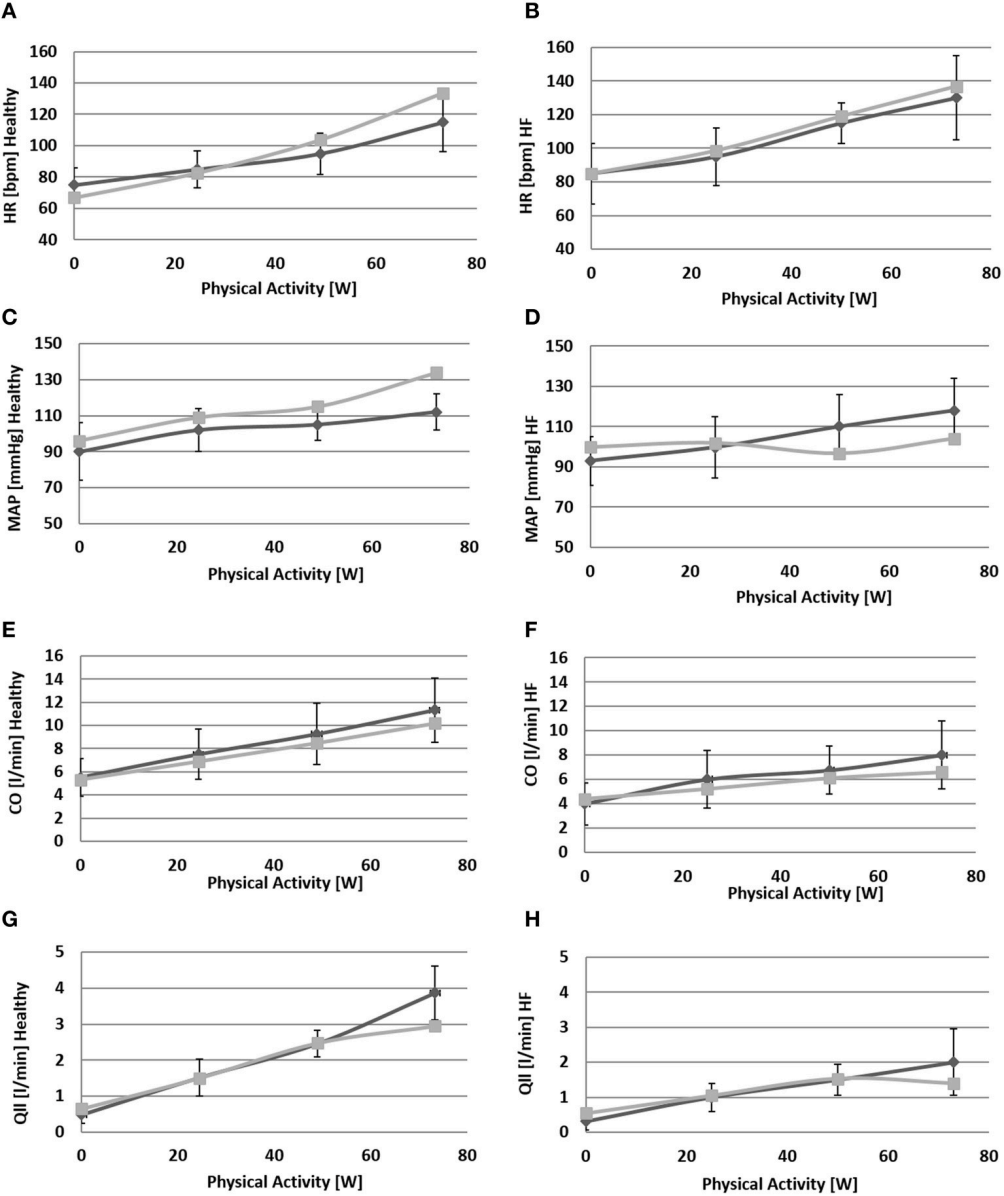
**Comparison between simulations output (light gray) and the data (light gray) from Sullivan et al. (1989)**. Left panels refer to healthy condition and right panels to heart failure condition. From **(A)** to **(H)**: heart rate (*HR*), mean arterial pressure (*MAP*), total cardiac output (*CO*), single leg flow (*Qlla*).

**Figure 7:**
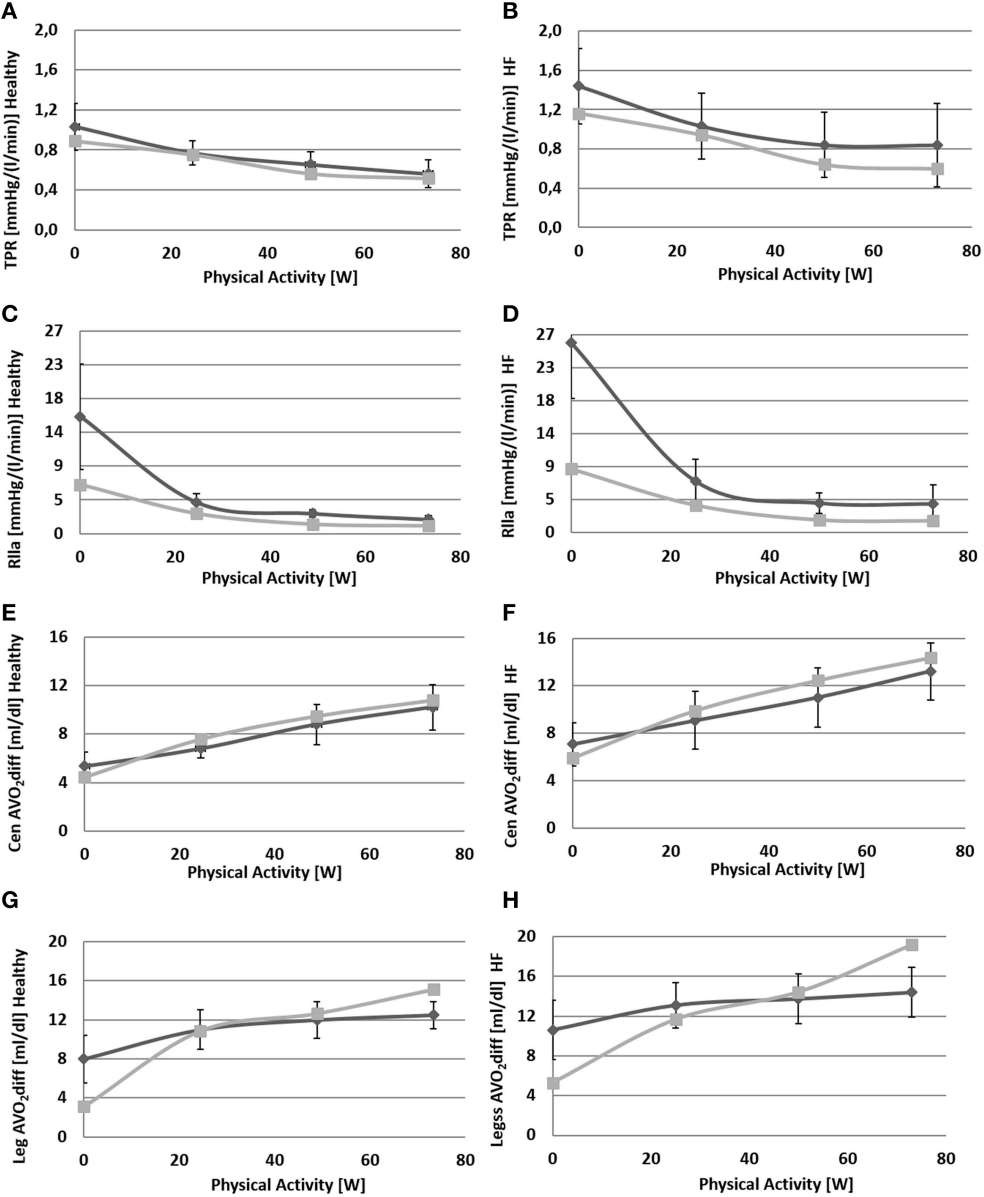
**Comparison between simulations output (light gray) and data (dark gray) from Sullivan et al. (1989)**. Left panels refer to healthy condition and right panels to heart failure condition. From **(A)** to **(H)**: total peripheral resistance (*TPR*), single leg resistance (*Rlla*), central arteriovenous oxygen difference, leg arteriovenous oxygen difference.

Figures 6, 7 show simulation results for both *Healthy* and *HF* at rest, at a workload of 24.5, 49, and 73 watts. In the text we will refer only to results at rest and at 73 watts, for brevity.

Due to baroreflex resetting *HR* increases for both *Healthy* (67–134 bpm) and *HF* (85–137 bpm). Total peripheral resistance decreases from 0.9 to 0.5 mmHg/(cm^3^/s) in *Healthy* and from 1.2 to 0.6 mmHg/(cm^3^/s) in *HF*. This is mainly due to the vasodilation of the lower limbs induced by the metabolic control. Single leg resistance, in fact, decreases from 6.5 to 1.0 mmHg/(cm^3^/s) for *Healthy* and from 8.7 to 1.6 mmHg/(cm^3^/s) for *HF*.

All these phenomena contribute to accommodating a higher *CO* during exercise: from 5.3 to 10.2 l/min in *Healthy* and from 4.4 to 6.6 l/min in *HF*. This increase is mostly addressed to better perfuse the legs. Single leg blood flow increases both in *Healthy* (0.7–3.0 l/min) and in *HF* (0.6–1.4 l/min). In terms of percentage, the flow of both legs is 26% of CO at rest and 59% during exercise in *Healthy*. For *HF* blood flow is 25% at rest and 42% during exercise.

The change in *TPR* and in *CO* also affects mean systemic arterial pressure (*MAP*): we observe an increment of *MAP* in *Healthy* (92–134 mmHg) while for *HF*, pressure attains at a rather constant value.

Figure 7 also provides some data about the ventilation section. The increment of oxygen uptake is reflected in the central arteriovenous oxygen difference that rises from 4.5 ml/dl to 10.8 ml/dl in *Healthy* and from 5.9 to 14.4 ml/dl in *HF*. Lower limbs show the highest augmentation in arteriovenous oxygen difference: from 3.1 to 15.1 ml/dl in *Healthy* and 5.3–19.2 ml/dl in *HF*.

**Figure 8:**
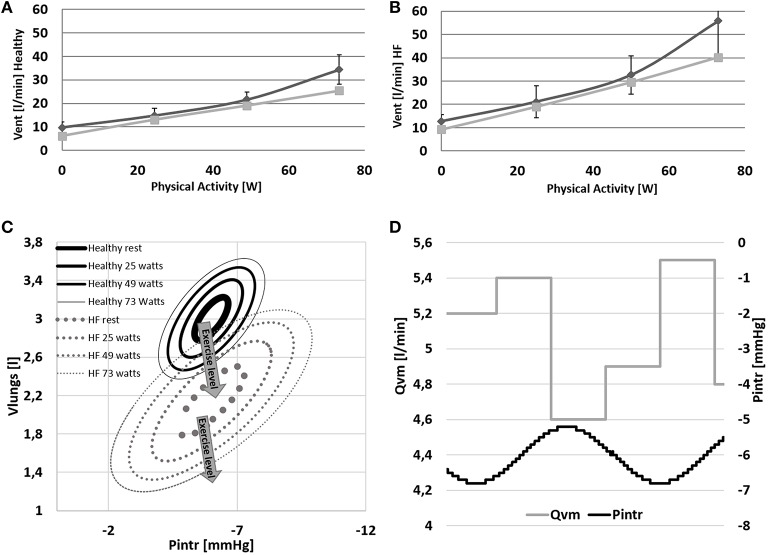
**(A,B)** Comparison between simulations output (light gray) and data (dark gray) from Sullivan et al. (1989). Data refer to ventilation in *Healthy* and *HF* conditions. **(C,D)** Pressure volume loop of the ventilation system at rest and at 24–48–73 watts of workload. Lower right panel: example of the effect of the intrathoracic pressure profile (*Pintr*) on mean venous return (*Qvm*).

Ventilation data are shown in Figure 8. In *Healthy* condition, ventilation increases from 6.1 to 25.5 l/min (Figure 8A), and in *HF* an even higher increase is observed (9.2–40.2 l/min, Figure 8B).

The change of ventilation pattern from rest to exercise is shown in Figure 8C. During exercise ventilation raises with a consequent increase of tidal volume and a deepening of *Pintr* during inspiration.

The mechanical effect of ventilation on venous return is shown in Figure 8D. During inspiration *Pintr* decreases thus improving venous return, the opposite effect is observed during expiration.

## Discussion

The cardiorespiratory simulator is composed of a cardiovascular model (Fresiello et al., 2015) integrated with respiratory and gas exchange models. Exercise was simulated by augmenting O_2_ uptake in specific regions, differentiating among exercising and non-exercising ones. Three regulations were implemented: the baroreflex, the metabolic and the ventilation controls.

The simulator shares some aspects with the one developed by Magosso and Ursino (2002): it provides a representation of exercise and resting vascular regions, baroreflex and metabolic regulations and the effect of muscle contraction on venous pressure.

The present work includes all these mechanisms plus some others. As we wanted to reproduce *HF* condition, we implemented a more sophisticated cardiovascular system, further developing some of the elements introduced by Magosso and Ursino (2002). In addition, we included the effect, gas exchange and the ventilation control that permitted the simulation of respiratory patterns and local arteriovenous oxygen differences.

The simulator does not provide a description of the overall human physiology (as in the case of Hester et al., 2011), but focuses only on those mechanisms that play a key role in exercise performance. This reduces the complexity of the overall structure, minimizing the number of equations and parameters. Such a simplification makes the simulator more easily adaptable for future research aimed at representing patients' specific conditions. An example of model personalization was already developed for the cardiovascular system and presented in Fresiello et al. (2015). As a future application, the proposed simulator will be used to reproduce a patient's specific hemodynamic and ventilation status, both at rest and exercise conditions.

The simulator reproduces the main cardiorespiratory changes observed during exercise for both *Healthy* and *HF*. The latter required a new parameter assignment for the cardiovascular, respiratory, baroreflex, and metabolic sub-models.

For *HF* we simulated a systolic impairment by reducing the *Elmax* parameter. The diastolic impairment, typical in chronic heart failure, was introduced by changing the filling characteristic. We also reproduced systemic and pulmonary hypertension by increasing the corresponding resistances (see Table 6 for more details).

For the baroreflex resetting we implemented the change of set-point pressure, the sympathetic overstimulation and the vagal withdrawal. All these mechanisms provoke heart chronotropy and inotropy and vasoconstriction for concurrent increasing values of aortic pressure. For *HF* the inotropic effect of baroreflex is less pronounced, since ventricular scar tissue has no capability to improve its contractility. This effect was simulated with a reduced sympathetic control gain on *Emaxl* (see Table 5, parameter *C*_*Elmaxs*_). As a consequence, left ventricular contractility increases less in *HF* (1.5–1.7 mmHg/cm^3^) than in *Healthy* (2.5–2.9 mmHg/cm^3^). Baroreflex also regulates the amount of blood stored in venous vessels thus contributing to the augmentation of cardiac output. During exercise, the sympathetic nervous system provokes a splanchnic arteriolar vasoconstriction and reduces venous capacity. As a result, a certain amount of blood is transferred from the splanchnic region to the large vessels and then to the heart (Laughlin et al., 2011). This mechanism was represented in our simulator through the sympathetic control of venous tone (*Vsp0*). As a final result we obtained a blood shift from the splanchnic region to the rest of the circulation of 287 cm^3^ in *Healthy* and of 206 cm^3^ in *HF*.

The increase in oxygen uptake lowers venous oxygen content such that the arteriovenous oxygen difference increases (see Figures 7E–H). This triggers the metabolic vasodilation especially in the exercising regions, where more blood needs to be supplied. The metabolic control shows a different behavior in *Healthy* and *HF*. In *HF* patients in fact, the chronic exposure of peripheral vessels to lower oxygen saturations makes the metabolic control less efficient. We reproduced this phenomenon by simply setting a lower *C*_*O*2*ivRef*_ in *HF*. This resulted in a reduced vasodilation during exercise, a lower perfusion, and higher arteriovenous oxygen difference in the exercising regions (see Figures 6H, 7D–H).

We also reproduced the sympatholysis effect, so that when a circulatory district exhibits a higher metabolic activity, the sensitivity of this region to sympathetic control is reduced. The interaction of sympathetic and metabolic regulations is fundamental for blood pressure and *CO*. The sympathetic outflow evokes peripheral vasoconstriction preventing a hypotension phenomenon, while the metabolic control improves perfusion where it is needed. The balance between these two mechanisms determines the final value of total peripheral resistance and the repartition of *CO* between exercising and non-exercising regions. Blood flow in the resting end organs is different between *Healthy* and *HF*: it stays rather constant in *Healthy* (4.0–4.2 l/min) and increases in *HF* (3.3–3.8 l/min). The reason for this opposite phenomenon, penalizing legs perfusion in *HF*, lies in the different regulation of peripheral resistances in resting regions. Combining upper body, kidneys and splanchnic districts we obtain an overall resistance at rest of 1.2 mmHg·s/cm^3^ for *Healthy* and 1.6 mmHg·s/cm^3^ for *HF*. At exercise we observe a vasoconstriction in *Healthy* (1.6 mmHg·s/cm^3^) while a slight vasodilation is observed for *HF* (1.4 mmHg·s/cm^3^).

From the model's point of view, this difference might reside in the lower central arteriovenous oxygen difference observed in *HF* during exercise (Figures 7E,F). This might have strengthened the metabolic vasodilation response of resting regions, preventing the sympathetic vasoconstriction from reducing oxygen supply. Similarly (Sullivan et al., 1989) report that *HF* patients, suffering from a low perfusion already at rest, show an increase of resting regions' blood flow to avoid possible ischemia in vital organs.

Some other differences between *Healthy* and *HF* are also observed at the different respiratory levels. *HF* shows a higher ventilation already at rest condition (6.1 l/min for *Healthy* and 9.2 l/min for *HF*). This difference increases even further with exercise (25.5 l/min for *Healthy* and 40.2 l/min for *HF*). The higher ventilation response in *HF* is the result of the increased *RQ* and of the reduced perfusion of ventilated lungs (Wasserman et al., 1997). The first phenomenon is due to the buffering of the accumulated lactic acid during exercise. Its representation goes beyond the aim of the present work but Equations (31) and (34) permit some consideration of this effect (*RQ* = 1.32 for *HF* and *RQ* = 0.96 for *Healthy* at *WL* = 73 watts). The reduced perfusion of ventilated lungs in *HF* was reproduced with a higher dead volume of the airways. This parameter was quantified according to Wasserman et al. (1997) reporting data of both healthy and heart failure subjects. Finally, the higher lungs elastance used for *HF* simulations, permitted to mimic an increased resistance to volume expansion. As a consequence, in *HF* a wider change of intrathoracic pressure is needed to obtain similar tidal volumes of *Healthy* (see Figure 8C).

With regard to the quality of simulations, we obtained a good match between our results and the data in the literature. The highest error was observed for legs parameters in both *Healthy* and *HF*. This discrepancy is due to a different value of the initial resistance at rest fed to the simulator (estimated from total peripheral resistance as reported in Fresiello et al., 2015), and the one of Sullivan et al. (1989). The difference between simulated and literature legs arteriovenous O_2_ difference (see Figure 7H) at *WL* = 73 is probably due to the anaerobic effect which was not taken into account as it goes beyond the scope of the present work.

In *HF* condition, all the phenomena described above and the relative impairments lead to a reduced capability to increase cardiac output adequately and to provide a sufficient perfusion to the exercising regions.

The present simulator can reproduce exercise capacity in *Healthy* and the basic pathophysiological mechanisms, limiting exercise capacity in *HF*. As a next step the simulator will be used to reproduce some diseases such as valve insufficiency, anemia, muscle tone impairment, chronotropic incompetence etc. The simulator will be used to evaluate how these diseases impair exercise and its related hemodynamic and ventilation parameters. The simulator will be used also to reproduce some therapies used in heart failure condition (i.e., medication, ventricular assist devices) and predict their effects on exercise capacity.

## Study limitations

The present simulator provides an overview of the main mechanisms occurring during aerobic exercise. Some simplifications were introduced to the model, as explained below.

At present the mechanisms leading to baroreflex resetting at the afferent level and their mutual interaction are not completely understood (Bevegård and Shepherd, 1996; Potts and Mitchell, 1998). The authors therefore implemented the resetting phenomenon directly at the efferent level.

The metabolic control model does not take into account the effects of different metabolites (other than hypoxia) on vasodilation during exercise (Pawelczyk et al., 1992). A more detailed metabolic control could better reproduce the arteriovenous oxygen difference in the legs for higher levels of exercise in *HF*, when anaerobic exercise occurs.

The model of pulmonary circulation is rather simple and does not include O_2_ and CO_2_ effects on vascular tone. Its simple structure permits an easy match with the implemented respiratory system which is also a simplified version including only one chamber, with no gravity ventilation-perfusion mismatch effect. Further improvements need to be implemented in order to get better simulation results, especially in terms of ventilation at higher levels of exercise.

## Conclusions

The proposed simulator permits the reproduction of the main physiological phenomena occurring during exercise at the level of cardiocirculatory and respiratory systems:

- cardiac output increase and its distribution among exercising and non-exercising regions,
- increase of heart activity and of vascular tone due to baroreflex resetting,
- peripheral resistance changes as a result of the combination of metabolic and baroreflex controls,
- central and local arteriovenous oxygen difference,
- increase of ventilation due to O_2_ and CO_2_ partial pressure changes during exercise.

Moreover, the simulator can reproduce heart failure condition, the related impairment of control mechanisms and their effects on exercise performance. The present simulator is suitable for such future applications as the representation of end-stage heart failure patients and the impact of therapies (such as drugs and ventricular assist devices) on their exercise performance.

## Author contributions

LF conception and design of the work, data collection, analysis and interpretation, manuscript drafting, final approval of the version to be published, agreement to be accountable for all aspects of the work in ensuring that questions related to the accuracy or integrity of any part of the work are appropriately investigated and resolved; BM, AD data interpretation, critical revision of the paper, final approval of the version to be published, agreement to be accountable for all aspects of the work in ensuring that questions related to the accuracy or integrity of any part of the work are appropriately investigated and resolved; GF supervision of work organization and development, data interpretation for important intellectual content, final approval of the version to be published, agreement to be accountable for all aspects of the work in ensuring that questions related to the accuracy or integrity of any part of the work are appropriately investigated and resolved.

### Conflict of interest statement

The authors declare that the research was conducted in the absence of any commercial or financial relationships that could be construed as a potential conflict of interest. The reviewer WAP and handling Editor declared a common affiliation and the handling Editor states that the process nevertheless met the standards of a fair and objective review.
