# Supplementary material for: A Model of the Cardiorespiratory Response to Aerobic Exercise in Healthy and Heart Failure Conditions
Source: Front Physiol. 2016 Jun 8;7:189. doi: 10.3389/fphys.2016.00189 (PMC4896934; doi:10.3389/fphys.2016.00189)
Supplement: Supplementary file 1 [file DataSheet1.pdf]

APPENDIX

Gas exchange equation.

Gas exchange was simulated with a mass balance equation between alveolar gas space and tissue (Longobardo et al., 1996, Betzel et al., 2007). We assume to have one homogeneous alveolar compartment perfused by pulmonary blood flow. The gas exchange at inspiration and expiration will be:

Inspiration

$$\frac{d}{dt}\Big((V_{E\exp}+V_A(\mathrm{t}))\cdot F_{O_2\,alv}(t)+Vap(t)\cdot C_{O_2\,ap}(\mathrm{t})\Big)=\dot{V}_A(t)\cdot F_{O_2 I}+(1-\mathrm{ps})\cdot\Big(Qpa(t)\cdot C_{O_2\,ap}(t)-Qpv(t)\cdot C_{O_2\,vp}(t)\Big)$$

Expiration

$$\frac{d}{dt}\Big((V_{Einsp}+V_A(t))\cdot F_{O_2\,alv}(t)+Vap(t)\cdot C_{O_2\,ap}(t)\Big)=-\dot{V}_A(t)\cdot F_{O_2\,alv}(t)+(1-\mathrm{ps})\cdot\Big(Qpa(t)\cdot C_{O_2\,ap}(t)-Qpv(t)\cdot C_{O_2\,vp}(t)\Big)$$

(1)

Where *Vap* is the pulmonary arterial volume, V_{Eexp} (V_{Einsp}) is the alveolar volume at the end of expiration (inspiration), V_A is the incremental alveolar volume, $\dot{V}_A$ is the alveolar ventilation over time calculated from *dVlungs/dt* in equation (4), subtracting the dead space ventilation calculated from equation (12). *Qpa* (*Qpv*) is the pulmonary arterial (venous) blood flow, *ps* is the pulmonary shunt, C_{O2ap} (C_{O2vp}) is the O_2 concentration in the arterial (venous) pulmonary blood, F_{O2alv} (F_{O2I}) is the molar fraction of O_2 in the alveoli (inspired air). Since ventilator flows and volumes are reported in BTPS units while blood gasses concentrations are expressed in STPD units a conversion needs to be made:

$$V_{BTPS}=\frac{P_{STPD}\cdot T_{BTPS}}{P_{BTPS}\cdot T_{STPD}}\cdot V_{STPD}=\frac{863}{(\mathrm{P_{amb}}-\mathrm{P_{H20}})}\cdot V_{STPD}$$

(2)

Using Dalton's law we can express the molar fraction in terms of partial pressure:

$$F_{O2}=\frac{P_{O2}}{P_{amb}-P_{H20}}$$

(3)

This leads to the following equations:

Inspiration

$$\frac{d}{dt}\Big((V_{E\exp}+V_A(t))\cdot P_{O_2\,alv}(t)+863\cdot Vap(t)\cdot C_{O_2\,ap}(t)\Big)=\dot{V}_A(t)\cdot P_{O_2 I}+863\cdot(1-\mathrm{ps})\cdot\Big(Qpa(t)\cdot C_{O_2\,ap}(t)-Qpv(t)\cdot C_{O_2\,vp}(t)\Big)$$

Expiration

$$\frac{d}{dt}\left((V_{Einsp}+V_A(t))\cdot P_{O_2\,alv}(t)+863\cdot Vap(t)\cdot C_{O_2\,ap}(t)\right)=-\dot{V}_A(t)\cdot P_{O_2\,alv}(t)+863\cdot(1-\mathrm{ps})\cdot\left(Qpa(t)\cdot C_{O_2\,ap}(t)-Qpv(t)\cdot C_{O_2\,vp}(t)\right) \quad (4)$$

Solving the differential equation and considering that:

$$\frac{dV_A(t)}{dt}=\begin{cases}\dot{V}_A(t) & \textit{for inspiration}\\ -\dot{V}_A(t) & \textit{for expiration}\end{cases}$$
$$\frac{dVap(t)}{dt}=(1-\mathrm{ps})\cdot Qvp(t)-Qap(t) \quad (5)$$

We finally obtain:

Inspiration

$$\left((V_{E\exp}+V_A(t))+863\cdot Vap(t)\cdot\frac{dC_{O_2ap}(t)}{dP_{O_2alv}(t)}\right)\cdot\frac{dP_{O_2alv}(t)}{dt}=863\cdot(1-ps)\cdot Qpv(t)\cdot\left(C_{O_2ap}(t)-C_{O_2vp}(t)\right)+\dot{V}_A(t)\cdot(P_{O_2I}(t)-P_{O_2alv}(t))$$

Expiration

$$\left((V_{Einsp}+V_A(t))+863\cdot Vap(t)\cdot\frac{dC_{O_2ap}(t)}{dP_{O_2alv}(t)}\right)\cdot\frac{dP_{O_2alv}(t)}{dt}=863\cdot(1-ps)\cdot Qpv(t)\cdot\left(C_{O_2ap}(t)-C_{O_2vp}(t)\right) \quad (6)$$

We assume that O_2 and CO_2 partial pressures are equal in the alveoli and in the arterial blood. Therefore, dC_{O2ap}/dP_{O2alv} is the slope of the dissociation curve in the operating point (Chiari et al., 1997). We considered a constant value of 0.0001625 (ml/mmHg·ml) (Longobardo et al., 1966).
For the carbon dioxide similar equations can be used with the difference that P_{O2I} can be neglected. The slope of the CO2 dissociation curve is set to 0.0065 ml/(mmHg·ml) (Longobardo et al., 1966).
